# Socio-demographic, behavioural and psycho-social factors associated with depression in two Russian cities

**DOI:** 10.1016/j.jad.2021.04.093

**Published:** 2021-07-01

**Authors:** Sarah Cook, Lyudmila Saburova, Natalia Bobrova, Ekaterina Avdeeva, Sofia Malyutina, Alexander V. Kudryavtsev, David A. Leon

**Affiliations:** a Faculty of Epidemiology and Population Health, London School of Hygiene & Tropical Medicine, London, United Kingdom; b Department of Community Medicine, UiT, The Arctic University of Norway, Tromsø, Norway; c Institute of Philosophy and Law, Ural Branch of the Russian Academy of Sciences, Ekaterinburg, Russian Federation; d Research Institute of Internal and Preventive Medicine, Branch of Institute of Cytology and Genetics, Siberian Branch of the Russian Academy of Sciences, Novosibirsk, Russian Federation; e Novosibirsk State Medical University, Russian Ministry of Health, Novosibirsk, Russian Federation; f Northern State Medical University, Arkhangelsk, Russian Federation; g International Laboratory for Population and Health, National Research University Higher School of Economics, Russian Federation; h National Heart and Lung Institute, Imperial College London, London, United Kingdom

**Keywords:** Depression, Mental health, Russian Federation, Alcohol, Social inequalities, Life events, CFA, Confirmatory factor analysis, CFI, Comparative Fit Index, CI, Confidence Interval, ESSE-RF, Epidemiology of Cardiovascular Diseases in various regions of Russia representative Federation, HAPIEE, Health, Alcohol and Psychosocial factors in Eastern Europe, OR, Odds ratio, PHQ-9, Patient Health Questionnaire-9, RMSEA, Root mean square error of approximation, SD, Standard deviation, SE, Standard error, TLI, Tucker Lewis Index

## Abstract

**Background:**

Russia has a high burden of suicide and alcohol-attributable mortality. However there have been few studies of the epidemiology of depression.

**Methods:**

The study population was 5077 men and women aged 35-69 years from a cross-sectional population based survey in the cities of Arkhangelsk and Novosibirsk (2015-17). Moderate depression was defined as Patient Health Questionnaire-9 (PHQ-9) score≥10. Risk factors considered were socio-demographic factors (age, sex, marital status, living alone, education, employment status, financial constraints); health behaviours (smoking, alcohol use) and psycho-social factors (life events and social support).

**Results:**

After mutual adjustment for all other factors, there was evidence that PHQ-9≥10 was associated with sex (higher in women), financial constraints, employment status, being a non-drinker, problem drinking, smoking, not having enough people to confide in and the number of life events in the past 6 months. Employment status was more strongly associated in men (OR 1.84 (95%CI 1.17, 2.88)) than women (OR 1.15 95% CI 0.86, 1.55). The effect size was particularly striking for financial constraints (odd ratio over 3 times higher in those with not enough money for food and clothes compared to no financial constraints), problem drinking (OR 1.72 (1.12, 2.65) among drinkers with CAGE score of 2 and 2.25 (95% CI 1.42, 3.57) in those with score ≥3 compared to zero) and life events (85% higher odds in those experiencing one life event and over 4 times higher odds in those experiencing 3 or more life events) all of which demonstrated a dose-response with PHQ-9>=10.

**Limitations:**

The study was cross-sectional in nature therefore temporal relationships could not be assessed.

**Conclusions:**

We have identified here a range of risk factors for depression among the Russian general population consistent with findings from other populations. The strikingly strong association with financial constraints indicates the importance of social inequality for the burden of depression.

## Highlights

• Socio-demographic risk factors were sex, financial constraints and employment status.
• Behaviour factors associated were being a non-drinker, problem drinking and smoking.
• Psycho-social factors were not having enough people to confide in and life events.
• Large effect size and dose-response for financial constraints, problem drinking and life events.
• Employment status was more strongly related to depressive symptoms in men than women.

## Introduction

Depression is an important public health concern worldwide associated with substantial morbidity (Steel et al., 2014; Whiteford et al., 2013). In Russia depression has been shown to be prospectively associated with all-cause and cardiovascular disease mortality (Kozela et al., 2016). This is in the context of very high national rates of cardiovascular disease (Townsend et al., 2016). Relatively little has been published in the International literature investigating risk factors for depression in Russia, despite high levels of deaths from self-harm and alcohol use disorders(GBD 2016 Russian Collaborators, 2018) suggesting substantial burden of disease.

Previous population-based studies from Russia have found associations of depression with socio-economic factors(Shal'nova et al., 2014), particularly current economic situation(Averina et al., 2005; Ferlander et al., 2016; Hsieh, 2015; Nicholson et al., 2008; Shal'nova et al., 2014), smoking(Averina et al., 2005), alcohol use (Averina et al., 2005; Bell et al., 2014) and psycho-social factors such as social capital and work-related stress(Ferlander et al., 2016; Hsieh, 2015; Pikhart et al., 2004; Ruiz et al., 2019). Few studies have included participants from rural areas but one study conducted in 1995 recruiting participants from three villages in rural Udmurtia found a higher prevalence of depression in women and those who were divorced but no evidence for an association with education(Pakriev et al., 1998a). In this study there was a strong association between alcohol dependence and depression in men of Russian ethnicity, not women or men of Udmurt ethnicity (Pakriev et al., 1998b). Only one of the above studies considered the relative strength of association of risk factors across several domains. Averina et al found similar effect sizes for odds of depression with smoking, alcohol use disorders, poor nutrition, low salary, and being widowed after mutual adjustment (odds ratios from 1.4-1.8) and a particularly strong association with gender (over three times higher in women)(Averina et al., 2005).This study from the city of Arkhangelsk (North West Russia) was conducted in 2000. In the last two decades Russia has under gone substantial political, economic and social changes (Shkolnikov et al., 2019). It is therefore timely to investigate the current relationship between a range of risk factors and depression in present day Russia and the relative strength of these different risk factors to identify at risk groups and opportunities for both prevention and intervention. The association of alcohol use and depression is of particular interest given high levels of alcohol-related mortality in Russia(Leon et al., 2007; Zaridze et al., 2009; Zaridze et al., 2014).

The Patient Health Questionnaire 9 (PHQ-9) is a validated tool for measuring depression(Kroenke et al., 2001) increasingly used within epidemiological studies, although it is not yet validated for use in Russia. Factor analysis of the PHQ-9 in a variety of populations has suggested a possible 2 factor structure with one factor for somatic symptoms and one for cognitive-affective symptoms (Boothroyd et al., 2019; Chilcot et al., 2013; Gonzalez-Blanch et al., 2018; Guo et al., 2017; Richards, 2008) although these are highly correlated(Boothroyd et al., 2019; Gonzalez-Blanch et al., 2018). The factor structure of the PHQ-9 has not been investigated within a Russian population sample.

The aim of this study was to investigate relative effect size of factors (demographic, socio-economic, psycho-social factors and health behaviours) associated with symptoms of depression in the Russian adult population in a recent general population survey (2015-17) conducted in two Russian cities and whether these are the same in men and women. We also investigate here the factor structure of the PHQ-9 instrument within a Russian population sample.

## Methods

The study population was men and women aged 35-69 participating in cross-sectional surveys of the general population in the Russian cities of Arkhangelsk and Novosibirsk (Know Your Heart)(Cook et al., 2018).

Participants were selected at random from population lists of addresses using stratified sampling by age and sex. Trained interviewers visited addresses and if participants agreed to take part in the study conducted a face to face interview. In the majority of cases this was done in the participant's home. The interview included questions on symptoms of depression, health behaviours and psycho-social factors. Among addresses where it was established that a participant of the correct age and sex was resident the response rate was 68% for Arkhangelsk and 41% for Novosibirsk(Cook et al., 2018).

### Outcome

The main outcome was moderate depression measured using the Patient Health Questionnaire 9 (PHQ-9)(Kroenke et al., 2001) from a standard Russian translation(Pfizer, 2002-2019). This is a nine-item scale with questions on symptoms of depression in the past 2 weeks. Each question has four response options (not at all/several days/more than half of the days/nearly every day). A severity score was calculated by summing responses to each question with “not at all” responses scored as zero and “nearly every day” as three. The standard cut point of PHQ-9 ≥10 was used to define moderate depression(Kroenke et al., 2001). Reporting of depression symptoms was used over reported previous history of depression due to very low levels of diagnosis and treatment of depression in the study population.

An alternative case definition(Kroenke et al., 2001) using an algorithm for identifying probable major depression was used for sensitivity analysis. Major depression was defined as present if a participant reported five or more of the nine symptoms at least “more than half the days” or more frequently and either anhedonia (question 1) or low mood (question 2) were included within the five symptoms. The exception to this was question 9 “thoughts that you would be better off dead or hurting yourself in some way” which was considered present if reported at all regardless of frequency.

### Exposures

The exposures of interest were demographic factors (age, sex, marital status, living alone) socio-economic factors (education, employment status and self-perceived financial constraints); health behaviours (smoking and alcohol use) and psycho-social factors (social support and number of life events).

Employment status was categorised as in regular paid employment or not. Education was grouped into three categories (lower than secondary, secondary (specialised secondary or vocational) and tertiary (incomplete higher and higher)). Financial constraints measured on 5 item likert scale reporting financial constraints ranging from “Not enough money for food” to “Enough money for a new car or better”.

Smoking status was categorised into never smoker, ex-smoker and current smoker. Alcohol use was measured by volume of ethanol consumed in the past 12 months calculated from beverage specific questions on the frequency and usual volume of ethanol consumed per occasion from beer, wine and spirits and from the CAGE score adapted to use a 12 month reference period(Mayfield et al., 1974). Psycho-social factors were measured from binary questions “Do you have people that you can confide in, to talk about personal matters when you need it?” and “Do you have people who can help you materially when you need it, e.g. pick up a child from school, take you to hospital?” and the number of life events in the past 6 months measured from The List of Threatening Experiences (Brugha et al., 1985; Brugha and Cragg, 1990).

### Analysis

*Psychometric properties of the PHQ-9 in the study population*

The PHQ-9 has not been formerly validated for use in Russia therefore we also considered some of its psychometric properties in our study population by calculating Cronbach's alpha and investigating the factor structure of the scale.

Factor structure of the scales was investigated by fitting three alternative confirmatory factor analysis (CFA) models using Weighted Least Squares with mean and variance adjusted and comparing model fit statistics and factor loadings.

Model 1) One factor model (manifested by all questions of PHQ-9).

Model 2)Two factors for depression (somatic factor manifested by 3 questions on somatic symptoms – sleeping problems, low energy, appetite; cognitive-affective factor manifested by other 6 questions)

Model 3) A bifactor structure with one general factor (manifested by all questions) and two specific factors (somatic and cognitive-affective factors as defined above) using Bi-Geomin rotation.

Model fit was assessed using the Comparative Fit Index (CFI), the Tucker Lewis Index (TLI) and the Root Mean Square Error of Approximation (RMSEA). CFI and TLI values greater than 0.95 indicate acceptable model fit( Tabachnik and Fidell, 1996) and for the RMSEA less than 0.08 indicates acceptable fit and less than 0.05 good fit(Streiner, 2006).

### Associations between depression and potential risk factors

The associations between the main outcome (PHQ-9≥10) and the exposures were assessed through separate logistic regression models with incremental adjustment for the different groups of variables 1) age, sex and city only 2) adjustment for all demographic and socio-economic variables and 3) further adjustment for health behaviours and psycho-social factors. For the demographic and socio-economic factors model 2 was considered the fully adjusted model but further adjustment for health behaviours and psycho-social factors was conducted to investigate the extent that these factors explained any associations. Interactions by sex and city were investigated by the use of likelihood ratio tests on the fully adjusted models with and without an interaction term for sex (or city) and each exposure (model 2 for socio-demographic factors; model 3 for health behaviours and psycho-social factors). Complete case analysis restricting the models to those with no missing data on any risk factors was used in order to compare odds ratios with and without adjustment.

Sensitivity analyses were conducted repeating analyses but using a lower cut off indicating mild depression (PHQ≥5)(Kroenke et al., 2001) and the algorithm for major depression to investigate the robustness of results to the case definition used for defining depression

Statistical analysis was conducted using Stata 16(StataCorp, 2017) and Mplus 7.31(Muthèn and Muthèn, 1998-2015)

### Ethical approval

All participants provided oral consent before completing the baseline questionnaire of the study and written consent after completing the questionnaire for this data to be used for the purposes of the study. This research complies with the Declaration of Helsinki. The Know Your Heart study was approved by the ethical committees of the London School of Hygiene & Tropical Medicine (approval number 8808 received 24/02/2015), Novosibirsk State Medical University (approval number 75 approval received 21/05/2015), the Institute of Preventative Medicine, Novosibirsk (no approval number; approval received 26/12/2014), and the Northern State Medical University, Arkhangelsk (approval number 01/01-15 received 27/01/2015). Ethical committees at each site subsequently approved use of the study data for secondary data analyses.

## Results

The study sample was 5077 participants aged 35-69 (42.8% men). The mean age was 54 years (SD 10). The prevalence of depression by severity score was 25.7% mild (5-9), 6.1% moderate (10-14) and 2.7% major (≥15). Using the algorithm for probable diagnosis of major depression the prevalence of depression was 3.1%.

Cronbach's alpha for the 9 items of the PHQ-9 was 0.77. The comparison of different factor structures for the PHQ-9 is shown in Table 1. All three models fitted the data very well with the best model fit for the 2 factor CFA model with separate but highly correlated somatic and cognitive-affective factors. The existing scoring system and cut points for the PHQ-9 are based on a unidimensional construct. Given the very high correlation between the two factors (0.87) we decided although there was evidence to support a two factor model the benefit from using a validated outcome for depression outweighed the likely benefit from using the two (highly correlated) factors separately therefore the tool was used as a unidimensional construct in the main analyses.

**Table 1:** Comparison of alternative factor structures of the PHQ-9.

			Model 1 CFA 1 Factor	Model 2 CFA 2 Factor		Model 3 Bi-factor 1 general factor and 2 specific factors		
**PHQ-9 Questions**			Factor 1Factor loading (SE)	Factor 1Factor loading (SE)	Factor 2Factor loading (SE)	Factor 1(General)Factor loading (SE)	Factor 2 (specific)Factor loading (SE)	Factor 3 (specific)Factor loading (SE)
Little interest or pleasure in doing things			0.75 (0.01)	0.76 (0.01)		0.71 (0.01)	-0.02 (0.01)	
Feeling down, depressed or hopeless			0.84 (0.01)	0.85 (0.01)		1.00 (0.00)	1.00 (0.00)	
Trouble falling or staying asleep, or sleeping too much			0.56 (0.01)		0.60 (0.01)	0.48 (0.01)		0.18 (0.01)
Feeling tired or having little energy			0.68 (0.01)		0.75 (0.01)	0.60 (0.01)		1.00 (0.00)
Poor appetite or overeating			0.53 (0.02)		0.57 (0.02)	0.49 (0.02)		0.11 (0.02)
Feeling bad about yourself- or that you are a failure or have let yourself or your family down			0.69 (0.01)	0.69 (0.01)		0.70 (0.02)	-0.10 (0.01)	
Trouble concentrating on things, such as reading the newspaper or watching television			0.68 (0.02)	0.69 (0.02)		0.71 (0.02)	-0.19 (0.01)	
Moving or speaking so slowly that other people have noticed. Or the opposite- being so fidgety or restless that you have been moving around a lot more than usual			0.65 (0.02)	0.65 (0.02)		0.68 (0.02)	0.22 (0.02)	
Thoughts that you would be better off dead or of hurting yourself in some way			0.69 (0.02)	0.70 (0.02)		0.71 (0.02)	-0.17 (0.02)	
Correlation between factors		Factor 1	-	-	-	-	0 (by constraint)	-
	Factor 2		-	0.87	-	-	0 (by constraint)	0 (by constraint)
	Factor 3		-					
Model Fit Statistics	CFI		0.97	0.98		0.98		
	TLI		0.96	0.97		0.97		
	RMSEA		0.058	0.051		0.053		

The prevalence of PHQ-9 score≥10 by demographic and socio-economic factors, health behaviours and psycho-social factors in shown in Tables 2 and 3. Sample size on restricting to those with no missing data on any risk factors was 4923.

**Table 2:** Association between demographic and socio-economic factors and PHQ-9≥10.

		PHQ-9≥10 n/N (%)		Model 1 OR (95% CI)*		Model 2 OR (95% CI)⁎⁎		Model 3 OR (95% CI)⁎⁎⁎	
Age	35-39	29/470	(6.2)	1.00	(ref)	1.00	(ref)	1.00	(ref)
	40-44	46/650	(7.1)	1.21	(0.74, 1.97)	1.16	(0.70, 1.90)	1.16	(0.69, 1.93)
	45-49	51/690	(7.4)	1.23	(0.76, 1.99)	1.22	(0.75, 2.00)	1.22	(0.74, 2.03)
	50-54	66/734	(9.0)	1.53	(0.96, 2.42)	1.39	(0.87, 2.23)	1.39	(0.85, 2.27)
	55-59	78/766	(10.2)	1.74	(1.10, 2.74)	1.40	(0.88, 2.24)	1.62	(0.99, 2.63)
	60-64	70/857	(8.2)	1.36	(0.86, 2.16)	0.93	(0.57, 1.51)	1.18	(0.71, 1.96)
	65+	109/910	(12.0)	2.07	(1.34, 3.20)	1.32	(0.83, 2.11)	1.88	(1.15, 3.08)
	Test for trend			P<0.001		P=0.59		P=0.21	
Sex	Male	125/2173	(5.8)	1.00	(ref)	1.00	(ref)	1.00	(ref)
	Female	324/2904	(11.2)	2.07	(1.66, 2.58)	1.91	(1.51,2.40)	2.74	(2.06, 3.65)
Marital status (missing=1)	Living with spouse	257/3483	(7.4)	1.00	(ref)	1.00	(ref)	1.00	(ref)
	Divorced/separated/widowed/never married	192/1593	(12.1)	1.44	(1.17, 1.78)	1.30	(1.02, 1.65)	1.14	(0.89, 1.45)
Lives alone (missing=13)	No	381/4462	(8.5)	1.00	(ref)	1.00	(ref)	1.00	(ref)
	Yes	66/602	(11.0)	1.11	(0.83, 1.48)	0.83	(0.60, 1.15)	0.92	(0.66, 1.28)
Education	Lower than secondary	46/377	(12.2)	1.50	(1.06, 2.11)	1.27	(0.89, 1.80)	1.24	(0.86, 1.78)
	Secondary	247/2676	(9.2)	1.00	(ref)	1.00	(ref)	1.00	(ref)
	Tertiary	156/2024	(7.7)	0.84	(0.67, 1.04)	0.97	(0.78, 1.21)	1.02	(0.81, 1.28)
	Test for trend			P=0.003		P=0.28		P=0.48	
Self-perceived financial constraints (missing=100)	Not enough for food	37/136	(27.2)	4.11	(2.72, 6.21)	3.54	(2.32, 5.39)	2.00	(1.27, 3.15)
	Enough for food but not clothes	125/887	(14.1)	1.58	(1.24, 2.01)	1.48	(1.16, 1.89)	1.18	(0.91, 1.52)
	Enough for food and clothes but difficult to buy large domestic appliances	213/2487	(8.6)	1.00	(ref)	1.00	(ref)	1.00	(ref)
	Enough for large domestic appliances but difficult to buy a new car	56/1201	(4.7)	0.58	(0.42, 0.78)	0.62	(0.45, 0.84)	0.72	(0.52, 0.99)
	Enough for a large car	9/26	(3.4)	0.45	(0.23, 0.89)	0.48	(0.24, 0.95)	0.52	(0.26, 1.05)
	Test for trend			P<0.001		P<0.001		P<0.001	
Employment status (missing=3)	In regular paid employment	198/2985	(6.6)	1.00	(ref)	1.00	(ref)	1.00	(ref)
	Not in regular paid employment	251/2089	(12.0)	1.79	(1.42, 2.26)	1.55	(1.22, 1.97)	1.29	(1.01, 1.64)

⁎ Model 1: Adjusted for age, sex and city

⁎⁎ Model 2: Model 1 + adjustment for all socio-demographic factors

⁎⁎⁎ Model 3: Model 2 + adjustment for smoking status, volume of ethanol, CAGE score and psycho-social factors; N for regression models= 4923

**Table 3:** Association between health behaviours and psycho-social factors and PHQ-9≥10.

		PHQ-9≥10 n/N (%)		Model 1 OR (95% CI)*		Model 2 OR (95% CI)⁎⁎		Model 3 (95% CI)⁎⁎⁎	
Smoking Status (Missing=2)	Never Smoker	208/2512	(8.3)	1.00	(ref)	1.00	(ref)	1.00	(ref)
	Ex-smoker	99/1178	(8.4)	1.48	(1.13, 1.95)	1.50	(1.14, 1.98)	1.41	(1.06, 1.88)
	Current Smoker	141/1385	(10.2)	1.98	(1.54, 2.55)	1.66	(1.28, 2.16)	1.43	(1.08, 1.88)
	Test for Trend			P<0.001		P<0.001		P=0.009	
Volume of ethanol (Missing=13)	Non drinker	139/1066	(13.0)	1.67	(1.32, 2.13)	1.47	(1.15, 1.87)	1.42	(1.11, 1.83)
	<2 Litres/year	197/2355	(8.4)	1.00	(ref)	1.00	(ref)	1.00	(ref)
	2-4.99 litres/year	41/699	(5.9)	1.02	(0.71, 1.47)	1.02	(0.70, 1.47)	0.86	(0.59, 1.26)
	5-9.99 Litres/year	38/490	(7.8)	1.50	(1.02, 2.22)	1.51	(1.02, 2.24)	1.28	(0.85, 1.93)
	10-19.99 Litres/year	17/280	(6.1)	1.22	(0.70, 2.13)	1.27	(0.72, 2.22)	1.03	(0.58, 1.82)
	>20 Litres/year	15/174	(8.6)	2.13	(1.18, 3.85)	1.66	(0.90, 3.04)	1.45	(0.78, 2.70)
	Test for Trend			P=0.63		P=0.95		P=0.39	
CAGE score among current drinkers	0	210/3054	(6.9)	1.00	(ref)	1.00	(ref)	1.00	(ref)
	1	41/486	(8.4)	1.87	(1.29, 2.72)	1.77	(1.21, 2.59)	1.53	(1.04, 2.27)
	2	36/330	(10.9)	2.66	(1.78, 3.98)	2.25	(1.49, 3.40)	1.72	(1.12, 2.65)
	3-4	36/300	(12.0)	3.80	(2.48, 5.82)	3.02	(1.94, 4.70)	2.25	(1.42, 3.57)
	Test for Trend			P<0.001		P<0.001		P<0.001	
Enough people to confide in (missing=20)	Yes	352/4494	(7.8)	1.00	(ref)	1.00	(ref)	1.00	(ref)
	No	95/563	(16.9)	2.49	(1.93, 3.22)	2.22	(1.71, 2.89)	2.00	(1.51, 2.66)
Enough people to help when needed (missing=20)	Yes	366/4462	(8.2)	1.00	(ref)	1.00	(ref)	1.00	(ref)
	No	83/590	(14.1)	1.90	(1.46, 2.47)	1.65	(1.26, 2.16)	1.14	(0.85, 1.54)
Number of life events in the past 6 months	0	111/2496	(4.5)	1.00	(ref)	1.00	(ref)	1.00	(ref)
	1	128/1407	(9.1)	2.13	(1.62, 2.78)	1.93	(1.47, 2.53)	1.85	(1.41, 2.44)
	2	98/669	(14.7)	3.42	(2.55, 4.59)	3.01	(2.23, 4.07)	2.81	(2.07, 3.80)
	3 or more	112/505	(22.2)	6.20	(4.62, 8.31)	4.86	(3.58, 6.59)	4.44	(3.26, 6.06)
	Test for trend			P<0.001		P<0.001		P<0.001	

⁎ Model 1: Adjusted for age, sex and city

⁎⁎ Model 2: Model 1 + adjustment for all socio-demographic factors

⁎⁎⁎ Model 3: Model 2 + mutual adjustment for all other variables in the table (volume of ethanol and CAGE score not adjusted for each other) N for regression models= 4923

### Association between Socio-demographic factors and Moderate Depression (PHQ-9≥10)

The associations between PHQ-9≥10 and demographic and socio-economic risk factors are shown in Table 2. After adjustment for age, sex and city there was strong evidence that the odds of PHQ-9≥10 were higher in participants who were women, not living with a spouse, less educated, with more financial constraints and not in regular paid employment. Living alone was not associated with PHQ-9≥10. There was strong evidence for a dose response with self-perceived financial constraints and education. After mutual adjustment for all socio-demographic factors there remained strong evidence for an association with sex, financial constraints, and employment status but not with age, marital status and education. The effect size was particularly large for financial constraints with those who reported not having enough money for food or clothes having 7 times higher odds of PHQ-9≥10 than those with very few financial constraints. Although attenuated the strong association with financial constraints (but not employment status) remained after further adjustment for health behaviours (alcohol use and smoking) and psycho-social factors and the effect size for sex differences increased (Table 2).

After mutual adjustment for all demographic and socio-economic factors there was good evidence (p=0.03) for an interaction between sex and employment status with a stronger association between employment status in men (OR 2.36 95% CI 1.52, 3.66) than in women (OR 1.32 95% CI 0.99, 1.77). After further adjustment for health behaviours and psycho-social factors the association between employment status and PHQ-9≥10 was only found in men (OR 1.84 (95% CI 1.17, 2.88) not women (OR 1.15 (95% CI 0.86, 1.55)) test for interaction p=0.07). There was no evidence for effect modification by sex or city for any other demographic or socio-economic risk factors.

### Association between Health Behaviours and Moderate Depression (PHQ-9≥10)

The associations between health behaviours (smoking status and alcohol use) with PHQ-9≥10 are shown in Table 3. There was strong evidence for higher odds of PHQ-9≥10 in both ex and current smokers compared to never smokers in all models. Odds were highest in current smokers after adjusting for socio-demographic factors, but after further adjustment for alcohol use and psycho-social factors the odds of moderate depression were approximately 40% higher than never smokers in both ex and current smokers.

After adjusting for age, sex and city there was a U-shaped relationship between volume of ethanol and odds of PHQ-9≥10 with higher odds in both non-drinkers and heavier drinkers. After adjusting for other socio-demographic factors the odds ratio for heavier drinkers became closer to the null but the increased odds in non-drinkers although attenuated remained after adjustment for both socio-demographic factors and further adjustment for smoking status and psycho-social factors. Among current drinkers there was strong evidence for higher odds of depression in problem drinkers with a dose-response with CAGE score. This remained after adjustment for all demographic, socio-economic, psycho-social factors and smoking.

There was no evidence for effect modification by sex or by city in the association between health behaviours and PHQ-9≥10.

### Association between Psycho-social factors and Moderate Depression (PHQ-9≥10)

The associations between psycho-social factors and PHQ-9≥10 are shown in Table 3. After adjustment for all demographic and socio-economic factors there was strong evidence for increased odds of depression in those reporting they did not have enough people to confide in and enough people to provide help when needed. There was strong evidence for a dose-response with the number of life events in the last 6 months with 93% higher odds in those experiencing one life event and over 4 times higher odds in those experiencing 3 or more life events. After mutual adjustment for the other psycho-social factors and health behaviours there remained strong evidence for associations with having enough people to confide in and life events.

There was no evidence of effect modification by sex or city on associations between psycho-social factors and PHQ-9≥10.

### Sensitivity Analysis using any depression (PHQ-9≥5) and Major depression (from algorithm) as alternative case definitions for the outcome

The results of sensitivity analysis using the lower cut point of PHQ-9≥5 are shown in Supplementary Tables 1 and 2. The pattern of association was similar overall but with some differences: 1) there was evidence for a trend in the odds of any depression with smoking even after adjusting for alcohol use and psycho-social factors; 2)the association with volume of ethanol consumed was U-shaped after minimal adjustment as with PHQ-9≥10, but after adjusting for all confounders there was no increased odds in non-drinkers while the odds in heavier drinkers remained higher; 3) the odds of any depression remained higher in those reporting they do not have enough people to help them even after adjustment for all confounders.

The results of sensitivity analyses using major depression derived using the algorithm are shown in Supplementary Tables 3 and 4. The results showed a very similar pattern of results as for PHQ-9≥10 but with stronger effect sizes for several risk factors (financial constraints, employment status, CAGE score and enough people to confide in and to help when needed).

## Discussion

This study provides the most comprehensive evidence on the association of a range of risk factors with depression in a contemporary population-based sample of the Russian population. After mutual adjustment for other factors included in the analysis, there was evidence that PHQ-9≥10 was associated with sex (higher in women), self-perceived financial constraints, employment status, being a non-drinker, problem drinking, smoking, not having enough people to confide in and the number of life events in the past 6 months. The effect size was particularly striking for financial constraints, problem drinking and life events all of which demonstrated a dose-response with moderate depression.

Our findings with respect to demographic factors are consistent with earlier studies from Russia and other countries. Higher levels of depression in women have been found almost universally both in Russia (Averina et al., 2005; Bobak et al., 2006; Pakriev et al., 1998a) and worldwide (Piccinelli and Wilkinson, 2000; Steel et al., 2014). We considered three measures of socio-economic position (education, employment status and financial constraints). There was a strong gradient with financial constraints but not education. The existing evidence on the association with education and depression from other countries is inconsistent. While several studies have found a protective relationship between higher education and depression(Domènech-Abella et al., 2018; Ross and Mirowsky, 2006) with some evidence for a stronger(Ross and Mirowsky, 2006) or exclusive effect in women(Arias-de la Torre et al., 2018) not all studies confirm this(Akhtar-Danesh and Landeen, 2007). Our findings are consistent with studies from several other countries showing financial strain is an important risk factor for depression(Dijkstra-Kersten et al., 2015; Domènech-Abella et al., 2018; Molarius et al., 2009; Weich and Lewis, 1998) and psychological distress(Tsuchiya et al., 2020). Our findings are also consistent with earlier studies from Russia (the HAPIEE study, Novosibirsk 2000)(Nicholson et al., 2008), The Arkhangelsk study,1999-2000(Averina et al., 2005) and The Moscow Health survey, 2004(Ferlander et al., 2016)) as well as the HAPIEE study populations in Poland and the Czech Republic(Nicholson et al., 2008) and the national ESSE RF study(Shal'nova et al., 2014) which found that measures of current financial or economic situation were more strongly associated with depression than education. We found here also evidence for a strong association with employment status in men but not in women (approximately two times higher odds of depression among those not in regular paid employment). A cross-country prospective study by the Organisation for Economic Co-operation and Development in five countries also found that overall mental health of men was more likely to be affected adversely by unemployment although findings were inconsistent between countries in terms of direction, size of effect and reason for unemployment (Organisation for Economic Co-operation and Development, 2008) which may reflect differences in perceptions of gender roles between societies. The gendered nature of Russian society where there are high levels of support for the view of men as the main source of financial support for a household(Motiejunaite and Kravchenko, 2008) may be an important factor in explaining the large difference in effect size between men and women found here. Our findings high-light the importance of addressing social inequalities, particularly current financial insecurity, in prevention of depression.

We found a strong link between depression and alcohol use disorders consistent with much research worldwide (Boden and Fergusson, 2011; Grant and Harford, 1995; Ross, 1995). Our findings are particularly relevant for Russia where hazardous alcohol consumption has been a major determinant of premature mortality (Leon et al., 2007; Zaridze et al., 2009; Zaridze et al., 2014) including deaths from suicide (Pridemore, 2013).Findings with regards to alcohol use were also consistent with previous findings from Russia: in the HAPIEE study 2003-5 odds of depression measured using Centre for Epidemiological Studies Depression Scale were 55% higher in abstainers and 66% higher in problems drinkers (CAGE score 2+) after controlling for confounding but depression symptoms were not associated with volume of ethanol (Bell et al., 2014). In the Arkhangelsk study 2000 odds of depression were 40% higher in hazardous drinkers and 80% higher in those with alcohol dependence as assessed by the Alcohol Use Disorders Identification Test (AUDIT)(Averina et al., 2005). We did not find here evidence for effect modification by sex in contrast to a study of 885 participants from rural Udmurtia in 1995 where a strong association with lifetime alcohol dependence was found in men of Russian ethnicity with an odds ratio close to three but no association was among women or men of Udmurt ethnicity, although findings from the earlier study should be treated with caution for comparisons here as they did not formally test for interaction or control for any confounders (Pakriev et al., 1998b). Abstainers are an atypical and heterogenous group including those who have stopped drinking due to previous alcohol problems or physical health problems. This may explain why odds of depression are higher in this group. In recent years alcohol consumption has been declining in Russia (Danilova et al., 2020; World Health Organisation, 2018) however levels of alcohol consumption still remain high (World Health Organisation, 2018) with alcohol use disorders the seventh largest cause of death in Russia in 2016(GBD 2016 Russian Collaborators, 2018). The findings shown here suggest the need to consider co-morbid depression in those with alcohol problems and the need to intervene on both to reduce mortality and improve quality of life in Russia.

We also found an association between smoking and depressive symptoms. This is consistent with a previous cross-sectional study of the general population in Russia (Averina et al., 2005). Similarly to alcohol use, the relationship between smoking and depression is well established but complex to understand with proposed bi-directional effects as well as confounding due to common causes. The evidence from observational studies remains inconclusive with varying results from different studies (Boden et al., 2010; Fluharty et al., 2017; Kendler et al., 1993; Munafò et al., 2008) however a recent Mendelian Randomisation study indicates that the relationship is unlikely to be causal(Taylor et al., 2014). To untangle these associations is beyond the scope of this paper but given historically high rates of smoking among Russian men (Giovino et al., 2012; Shkolnikov et al., 2020) it is important to identify the higher depression risk in this sub-group in the population.

Finally, we considered three psycho-social measures. Of these we found both not having enough people to confide in and the number of life events in the previous six months were strong independent risk factors for depression. It should be noted though that living alone was not associated with depression. Findings are consistent with research from Russia(Brailovskaia et al., 2018) and several other populations on the importance of social support (Alsubaie et al., 2019; Gariépy et al., 2016; Roohafza et al., 2014) and life events as risk factors for depression for example(Kendler et al., 1998; Kessler, 1997; Kinderman et al., 2013; Lueboonthavatchai, 2009; Molarius et al., 2009; You and Conner, 2009). What is particularly striking here is the larger effect size for number of life events in contrast with the other risk factors included in the analysis. This may be influenced by the time frame of measurement (past 6 months) as previous research has shown particularly large effect sizes for life events the more recent the event was experienced(Kendler et al., 1998).

This study has several limitations, which should be considered when interpreting the findings. First this was a cross-sectional study and we cannot make an inference about causality from these findings. Reverse causality and bi-directional relationships are possible for a number of the risk factors considered here for example depressive symptoms may increase levels of smoking and alcohol consumption and also impact on perception of social support. Furthermore, here we have used a measure of self-reported depression symptoms in a two-week period rather than clinically diagnosed depression. The PHQ-9 whilst a validated tool (Kroenke et al., 2001) has not been validated for use in Russia however we were able to demonstrate high internal reliability (Cronbach's alpha 0.77) and a two factor structure consistent with other populations (Boothroyd et al., 2019; Chilcot et al., 2013; Gonzalez-Blanch et al., 2018; Guo et al., 2017; Richards, 2008)

We have considered here a range of potential risk factors for depression covering several domains. However, this is not a comprehensive list of all possible risk factors and residual confounding due to exclusion of unmeasured confounders for the associations presented here is a possibility. Exposures were not constrained to have the same number of exposure categories in order to retain categories with a substantive meaning and odds ratios are not directly comparable across risk factors. Therefore while we were able to detect qualitatively risk factors with particularly strong effect sizes we have not made a formal assessment of the contribution of each risk factor. There is also the possibility of selection bias. The study population included participants from two cities in Russia, and the response rates for one of the study sites in particular were low. It is possible that we were less likely to include those with more extreme values of exposure and outcome (such as those with higher levels of depression, heavier drinkers, most disadvantaged financially) limiting the power to estimate precisely the associations studied here. However unless the association between risk factors and depression is different in our study population compared with non-responders the associations should be internally valid. We did not find here any evidence for interaction by city for any of the risk factors investigated which suggests the differential non-response between the cities did not impact on the study findings despite the fact that survey response rates differed.

Finally our findings may not be generalizable to the whole of Russia as we included here two cities and our study alongside the majority of previous studies of risk factors for depression in Russia is restricted to urban populations. Further work investigating the prevalence and risk factors for depression among rural Russian populations are needed given only one study from 1995 has studied risk factors for depression specifically in rural participants (Pakriev et al., 1998a; Pakriev et al., 1998b).

In conclusion here we have assessed the strength of associations with depression across four domains - demographic factors, socio-economic factors, health behaviours and psycho-social factors. The risk factors identified here are consistent with findings from other populations. We found strikingly strong associations with a factor from each domain – gender, financial constraints, problem drinking and recent life events. This is particularly relevant in Russia where psychiatric services have been traditionally focused on delivery of care from specialist outpatient psychiatric dispensaries or inpatient hospitalisation (Füredi et al., 2006; Gurovich, 2007; Jenkins et al., 2007) while depression is not commonly assessed or treated within primary care services (Gurovich, 2007; Jenkins et al., 2007; Rezvy et al., 2019; Sorgaard et al., 2013). Our findings are consistent with a bio-psycho-social model of depression and indicates the need to take a holistic approach in prevention and treatment of depression integrated within the health care system.

## Declaration of Competing Interest

None declared

## Appendix Supplementary materials

Image, application 1

## References

[bib0001] Akhtar-Danesh N., Landeen J. (2007). Relation between depression and sociodemographic factors. International Journal of Mental Health Systems.

[bib0002] Alsubaie M.M., Stain H.J., Webster L.A.D., Wadman R. (2019). The role of sources of social support on depression and quality of life for university students. International Journal of Adolescence and Youth.

[bib0003] Arias-de la Torre J., Vilagut G., Martín V., Molina A.J., Alonso J. (2018). Prevalence of major depressive disorder and association with personal and socio-economic factors. Results for Spain of the European Health Interview Survey 2014–2015. J. Affect. Disord..

[bib0004] Averina M., Nilssen O., Brenn T., Brox J., Arkhipovsky V.L., Kalinin A.G. (2005). Social and lifestyle determinants of depression, anxiety, sleeping disorders and self-evaluated quality of life in Russia–a population-based study in Arkhangelsk. Soc. Psychiatry Psychiatr. Epidemiol..

[bib0005] Bell S., Britton A., Kubinova R., Malyutina S., Pajak A., Nikitin Y., Bobak M. (2014). Drinking pattern, abstention and problem drinking as risk factors for depressive symptoms: evidence from three urban Eastern European populations. PLoS One.

[bib0006] Bobak M., Pikhart H., Pajak A., Kubinova R., Malyutina S., Sebakova H., Topor-Madry R., Nikitin Y., Marmot M. (2006). Depressive symptoms in urban population samples in Russia, Poland and the Czech Republic. Br. J. Psychiatry.

[bib0007] Boden J.M., Fergusson D.M. (2011). Alcohol and depression. Addiction.

[bib0008] Boden J.M., Fergusson D.M., Horwood L.J. (2010). Cigarette smoking and depression: tests of causal linkages using a longitudinal birth cohort. Br. J. Psychiatry.

[bib0009] Boothroyd L., Dagnan D., Muncer S. (2019). PHQ-9: One factor or two?. Psychiatry Res..

[bib0010] Brailovskaia J., Schonfeld P., Zhang X.C., Bieda A., Kochetkov Y., Margraf J. (2018). A Cross-Cultural Study in Germany, Russia, and China: Are Resilient and Social Supported Students Protected Against Depression, Anxiety, and Stress?. Psychol. Rep..

[bib0011] Brugha T., Bebbington P., Tennant C., Hurry J. (1985). The List of Threatening Experiences: a subset of 12 life event categories with considerable long-term contextual threat. Psychol. Med..

[bib0012] Brugha T.S., Cragg D. (1990). The List of Threatening Experiences: the reliability and validity of a brief life events questionnaire. Acta Psychiatr. Scand..

[bib0013] Chilcot J., Rayner L., Lee W., Price A., Goodwin L., Monroe B., Sykes N., Hansford P., Hotopf M. (2013). The factor structure of the PHQ-9 in palliative care. J. Psychosom. Res..

[bib0014] Cook, S., Malyutina, S., Kudryavtsev, A., Averina, M., Bobrova, N., Boytsov, S., Brage, S., Clark, T., Diez Benavente, E., Eggen, A., Hopstock, L., Hughes, A., Johansen, H., Kholmatova, K., Kichigina, A., Kontsevaya, A., Kornev, M., Leong, D., Magnus, P., Mathiesen, E., McKee, M., Morgan, K., Nilssen, O., Plakhov, I., Quint, J., Rapala, A., Ryabikov, A., Saburova, L., Schirmer, H., Shapkina, M., Shiekh, S., Shkolnikov, V., Stylidis, M., Voevoda, M., Westgate, K., Leon, D., 2018. Know Your Heart: Rationale, design and conduct of a cross-sectional study of cardiovascular structure, function and risk factors in 4500 men and women aged 35-69 years from two Russian cities, 2015-18 [version 3; peer review: 3 approved]. Wellcome Open Research 3. 10.12688/wellcomeopenres.14619.2 PMC6073094 30123849

[bib0015] Danilova I., Shkolnikov V.M., Andreev E., Leon D.A. (2020). The changing relation between alcohol and life expectancy in Russia in 1965–2017. Drug Alcohol Rev..

[bib0016] Dijkstra-Kersten S.M.A., Biesheuvel-Leliefeld K.E.M., van der Wouden J.C., Penninx B.W.J.H., van Marwijk H.W.J. (2015). Associations of financial strain and income with depressive and anxiety disorders. J. Epidemiol. Community Health.

[bib0017] Domènech-Abella J., Mundó J., Leonardi M., Chatterji S., Tobiasz-Adamczyk B., Koskinen S., Ayuso-Mateos J.L., Haro J.M. (2018). The association between socioeconomic status and depression among older adults in Finland, Poland and Spain: A comparative cross-sectional study of distinct measures and pathways. J. Affect. Disord..

[bib0018] Ferlander S., Stickley A., Kislitsyna O., Jukkala T., Carlson P., Makinen I.H. (2016). Social capital - a mixed blessing for women? A cross-sectional study of different forms of social relations and self-rated depression in Moscow. BMC Psychol.

[bib0019] Fluharty M., Taylor A.E., Grabski M., Munafo M.R. (2017). The Association of Cigarette Smoking With Depression and Anxiety: A Systematic Review. Nicotine Tob. Res..

[bib0020] Füredi J., Mohr P., Swingler D., Bitter I., Gheorghe M.D., Hotujac L., Jarema M., Kocmur M., Koychev G.I., Mosolov S.N., Pecenak J., Rybakowski J., Svestka J., Sartorius N. (2006). Psychiatry in selected countries of Central and Eastern Europe: An overview of the current situation. Acta Psychiatr. Scand..

[bib0021] Gariépy G., Honkaniemi H., Quesnel-Vallée A. (2016). Social support and protection from depression: systematic review of current findings in Western countries. Br. J. Psychiatry.

[bib0022] GBD 2016 Russian Collaborators (2018). The burden of disease in Russia from 1980 to 2016: a systematic analysis for the Global Burden of Disease Study 2016. Lancet.

[bib0023] Giovino G.A., Mirza S.A., Samet J.M., Gupta P.C., Jarvis M.J., Bhala N., Peto R., Zatonski W., Hsia J., Morton J., Palipudi K.M., Asma S. (2012). Tobacco use in 3 billion individuals from 16 countries: an analysis of nationally representative cross-sectional household surveys. Lancet North Am. Ed..

[bib0024] Gonzalez-Blanch C., Medrano L.A., Munoz-Navarro R., Ruiz-Rodriguez P., Moriana J.A., Limonero J.T., Schmitz F., Cano-Vindel A. (2018). Factor structure and measurement invariance across various demographic groups and over time for the PHQ-9 in primary care patients in Spain. PLoS One.

[bib0025] Grant B.F., Harford T.C. (1995). Comorbidity between DSM-IV alcohol use disorders and major depression: results of a national survey. Drug Alcohol Depend..

[bib0026] Guo B., Kaylor-Hughes C., Garland A., Nixon N., Sweeney T., Simpson S., Dalgleish T., Ramana R., Yang M., Morriss R. (2017). Factor structure and longitudinal measurement invariance of PHQ-9 for specialist mental health care patients with persistent major depressive disorder: Exploratory Structural Equation Modelling. J. Affect. Disord..

[bib0027] Gurovich I.Y. (2007). The Current Status of Psychiatric Services in Russia: Moving towards Community based psychiatry. International Journal of disability and community rehabilitation.

[bib0028] Hsieh N. (2015). Economic Security, Social Cohesion, and Depression Disparities in Post-transition Societies: A Comparison of Older Adults in China and Russia. J. Health Soc. Behav..

[bib0029] Jenkins R., Lancashire S., McDaid D., Samyshkin Y., Green S., Watkins J., Potasheva A., Nikiforov A., Bobylova Z., Gafurov V., Goldberg D., Huxley P., Lucas J., Purchase N., Atun R. (2007). Mental health reform in the Russian Federation: an integrated approach to achieve social inclusion and recovery. Bull. World Health Organ..

[bib0030] Kendler K.S., Karkowski L.M., Prescott C.A. (1998). Stressful Life Events and Major Depression: Risk Period, Long-Term Contextual Threat, and Diagnostic Specificity. J. Nerv. Ment. Dis..

[bib0031] Kendler K.S., Neale M.C., MacLean C.J., Heath A.C., Eaves L.J., Kessler R.C. (1993). Smoking and Major Depression: A Causal Analysis. Arch. Gen. Psychiatry.

[bib0032] Kessler R.C. (1997). The effects of stressful life events on depression. Annu. Rev. Psychol..

[bib0033] Kinderman P., Schwannauer M., Pontin E., Tai S. (2013). Psychological Processes Mediate the Impact of Familial Risk, Social Circumstances and Life Events on Mental Health. PLoS One.

[bib0034] Kozela M., Bobak M., Besala A., Micek A., Kubinova R., Malyutina S., Denisova D., Richards M., Pikhart H., Peasey A., Marmot M., Pajak A. (2016). The association of depressive symptoms with cardiovascular and all-cause mortality in Central and Eastern Europe: Prospective results of the HAPIEE study. Eur J Prev Cardiol.

[bib0035] Kroenke K., Spitzer R.L., Williams J.B.W. (2001). The PHQ-9: Validity of a brief depression severity measure. J. Gen. Intern. Med..

[bib0036] Leon D.A., Saburova L., Tomkins S., Andreev E., Kiryanov N., McKee M., Shkolnikov V.M. (2007). Hazardous alcohol drinking and premature mortality in Russia: a population based case-control study. Lancet.

[bib0037] Lueboonthavatchai P. (2009). Role of stress areas, stress severity, and stressful life events on the onset of depressive disorder: a case-control study. J. Med. Assoc. Thai..

[bib0038] Mayfield D., McLeod G., Hall P. (1974). The CAGE questionnaire: validation of a new alcoholism screening instrument. Am. J. Psychiatry.

[bib0039] Molarius A., Berglund K., Eriksson C., Eriksson H.G., Lindén-Boström M., Nordström E., Persson C., Sahlqvist L., Starrin B., Ydreborg B. (2009). Mental health symptoms in relation to socio-economic conditions and lifestyle factors – a population-based study in Sweden. BMC Public Health.

[bib0040] Motiejunaite A., Kravchenko Z. (2008). Family policy, employment and gender-role attitudes: a comparative analysis of Russia and Sweden. Journal of European Social Policy.

[bib0041] Munafò M.R., Hitsman B., Rende R., Metcalfe C., Niaura R. (2008). Effects of progression to cigarette smoking on depressed mood in adolescents: evidence from the National Longitudinal Study of Adolescent Health. Addiction.

[bib0042] Muthèn L.K., Muthèn B.O. (1998).

[bib0043] Nicholson A., Pikhart H., Pajak A., Malyutina S., Kubinova R., Peasey A., Topor-Madry R., Nikitin Y., Capkova N., Marmot M., Bobak M. (2008). Socio-economic status over the life-course and depressive symptoms in men and women in Eastern Europe. J. Affect. Disord..

[bib0044] Organisation for Economic Co-operation and Development (2008).

[bib0045] Pakriev S., Vasar V., Aluoja A., Saarma M., Shlik J. (1998). Prevalence of mood disorders in the rural population of Udmurtia. Acta Psychiatr. Scand..

[bib0046] Pakriev S., Vasar V., Aluoja A., Shlik J. (1998). Prevalence of ICD-10 harmful use of alcohol and alcohol dependence among the rural population in Udmurtia. Alcohol Alcohol..

[bib0047] Pfizer, 2002 -2019. www.phqscreeners.com.

[bib0048] Piccinelli M., Wilkinson G. (2000). Gender differences in depression: Critical review. Br. J. Psychiatry.

[bib0049] Pikhart H., Bobak M., Pajak A., Malyutina S., Kubinova R., Topor R., Sebakova H., Nikitin Y., Marmot M. (2004). Psychosocial factors at work and depression in three countries of Central and Eastern Europe. Soc. Sci. Med..

[bib0050] Pridemore W.A. (2013). The impact of hazardous drinking on suicide among working-age Russian males: an individual-level analysis. Addiction.

[bib0051] Rezvy G., Andreeva E., Ryzhkova N., Yashkovich V., Sørlie T. (2019). Integrating mental health into primary care in Arkhangelsk County, Russia: the Pomor model in psychiatry. International journal of mental health systems.

[bib0052] Richards J.S. (2008). Factor Structure of the PHQ-9 Screen for Depression Across Time Since Injury Among Persons With Spinal Cord Injury. Rehabilitation Psychology.

[bib0053] Roohafza H.R., Afshar H., Keshteli A.H., Mohammadi N., Feizi A., Taslimi M., Adibi P. (2014). What's the role of perceived social support and coping styles in depression and anxiety?. J Res Med Sci.

[bib0054] Ross C.E., Mirowsky J. (2006). Sex differences in the effect of education on depression: Resource multiplication or resource substitution?. Soc. Sci. Med..

[bib0055] Ross H.E. (1995). DSM-III-R alcohol abuse and dependence and psychiatric comorbidity in Ontario: results from the Mental Health Supplement to the Ontario Health Survey. Drug Alcohol Depend..

[bib0056] Ruiz M., Malyutina S., Pajak A., Kozela M., Kubinova R., Bobak M. (2019). Congruent relations between perceived neighbourhood social cohesion and depressive symptoms among older European adults: An East-West analysis. Soc. Sci. Med..

[bib0057] Shal’nova S.A., Evstifeeva S.E., Deev A.D., Artamova G.V., Gatagonova T.M., Dupliakov D.V., Efanov A., Zhernakova Iu V., Konradi A.O., Libis R.A., Minakov E.V., Nedogoda S.V., Oshchepkova E.V., Romanchuk S.V., Rotar O.P., Trubacheva I.A., EV S.H., Boitsov S.A. (2014). [The prevalence of anxiety and depression in different regions of the Russian Federation and its association with sociodemographic factors (according to the data of the ESSE-RF study)]. Ter. Arkh..

[bib0058] Shkolnikov V.M., Andreev E.M., Tursun-zade R., Leon D.A. (2019). Patterns in the relationship between life expectancy and gross domestic product in Russia in 2005–15: a cross-sectional analysis. The Lancet Public Health.

[bib0059] Shkolnikov V.M., Churilova E., Jdanov D.A., Shalnova S.A., Nilssen O., Kudryavtsev A., Cook S., Malyutina S., McKee M., Leon D.A. (2020). Time trends in smoking in Russia in the light of recent tobacco control measures: synthesis of evidence from multiple sources. BMC Public Health.

[bib0060] Sorgaard K.W., Rezvy G., Bugdanov A., Sorlie T., Bratlid T. (2013). Treatment needs, diagnoses and use of services for acutely admitted psychiatric patients in northwest Russia and northern Norway. International Journal of Mental Health Systems.

[bib0061] StataCorp (2017).

[bib0062] Steel Z., Marnane C., Iranpour C., Chey T., Jackson J.W., Patel V., Silove D. (2014). The global prevalence of common mental disorders: a systematic review and meta-analysis 1980–2013. Int. J. Epidemiol..

[bib0063] Streiner D.L. (2006). Building a better model: An introduction to structural equation modelling. Can J Psychiat.

[bib0064] Tabachnik B.G., Fidell L.S. (1996).

[bib0065] Taylor A.E., Fluharty M.E., Bjørngaard J.H., Gabrielsen M.E., Skorpen F., Marioni R.E., Campbell A., Engmann J., Mirza S.S., Loukola A., Laatikainen T., Partonen T., Kaakinen M., Ducci F., Cavadino A., Husemoen L.L.N., Ahluwalia T.S., Jacobsen R.K., Skaaby T., Ebstrup J.F., Mortensen E.L., Minica C.C., Vink J.M., Willemsen G., Marques-Vidal P., Dale C.E., Amuzu A., Lennon L.T., Lahti J., Palotie A., Räikkönen K., Wong A., Paternoster L., Wong A.P., Horwood L.J., Murphy M., Johnstone E.C., Kennedy M.A., Pausova Z., Paus T., Ben-Shlomo Y., Nohr E.A., Kuh D., Kivimaki M., Eriksson J.G., Morris R.W., Casas J.P., Preisig M., Boomsma D.I., Linneberg A., Power C., Hyppönen E., Veijola J., Jarvelin M.R., Korhonen T., Tiemeier H., Kumari M., Porteous D.J., Hayward C., Romundstad P.R., Smith G.D., Munafò M.R. (2014). Investigating the possible causal association of smoking with depression and anxiety using Mendelian randomisation meta-analysis: the CARTA consortium.. BMJ Open.

[bib0066] Townsend N., Wilson L., Bhatnagar P., Wickramasinghe K., Rayner M., Nichols M. (2016). Cardiovascular disease in Europe: epidemiological update 2016. Eur. Heart J..

[bib0067] Tsuchiya K., Leung C.W., Jones A.D., Caldwell C.H. (2020). Multiple financial stressors and serious psychological distress among adults in the USA. Int J Public Health.

[bib0068] Weich S., Lewis G. (1998). Poverty, unemployment, and common mental disorders: population based cohort study. BMJ.

[bib0069] Whiteford H.A., Degenhardt L., Rehm J., Baxter A.J., Ferrari A.J., Erskine H.E., Charlson F.J., Norman R.E., Flaxman A.D., Johns N., Burstein R., Murray C.J., Vos T. (2013). Global burden of disease attributable to mental and substance use disorders: findings from the Global Burden of Disease Study 2010. Lancet.

[bib0070] World Health Organisation (2018).

[bib0071] You S., Conner K.R. (2009). Stressful life events and depressive symptoms: influences of gender, event severity, and depression history. J. Nerv. Ment. Dis..

[bib0072] Zaridze D., Brennan P., Boreham J., Boroda A., Karpov R., Lazarev A., Konobeevskaya I., Igitov V., Terechova T., Boffetta P., Peto S. (2009). Alcohol and cause-specific mortality in Russia: a retrospective case-control study of 48 557 adult deaths. Lancet.

[bib0073] Zaridze D., Lewington S., Boroda A., Scelo G., Karpov R., Lazarev A., Konobeevskaya I., Igitov V., Terechova T., Boffetta P., Sherliker P., Kong X., Whitlock G., Boreham J., Brennan P., Peto R. (2014). Alcohol and mortality in Russia: prospective observational study of 151,000 adults. Lancet.

