# Supplementary material for: Socio-demographic, behavioural and psycho-social factors associated with depression in two Russian cities
Source: J Affect Disord. 2021 Jul 1;290:202–10. doi: 10.1016/j.jad.2021.04.093 (PMC8626563; doi:10.1016/j.jad.2021.04.093)
Supplement: Supplementary file 1 [file mmc1.docx]

**Supplementary Table 1. Association between demographic and socio-economic factors and PHQ-9≥5**

|  | | PHQ-9≥5 n/N (%) | | Model 1 OR (95% CI)* | | Model 2 OR (95% CI)** | | Model 3 OR (95% CI)*** | | | |
| --- | --- | --- | --- | --- | --- | --- | --- | --- | --- | --- | --- |
| Age | 35-39 | 158/470 | (33.6) | 1.00 | (ref) | 1.00 | (ref) | 1.00 | (ref) | | |
|  | 40-44 | 218/650 | (33.5) | 1.04 | (0.80, 1.35) | 1.00 | (0.77, 1.29) | 0.96 | (0.73, 1.26) | | |
|  | 45-49 | 220/690 | (31.9) | 0.95 | (0.73, 1.23) | 0.94 | (0.72, 1.22) | 0.90 | (0.69, 1.18) | | |
|  | 50-54 | 244/734 | (33.2) | 1.01 | (0.78, 1.30) | 0.95 | (0.73, 1.22) | 0.94 | (0.72, 1.23) | | |
|  | 55-59 | 284/766 | (37.1) | 1.20 | (0.93, 1.54) | 1.06 | (0.82, 1.37) | 1.14 | (0.87, 1.49) | | |
|  | 60-64 | 283/857 | (33.0) | 0.99 | (0.78, 1.27) | 0.80 | (0.62, 1.04) | 0.93 | (0.70, 1.22) | | |
|  | 65-69 | 350/910 | (38.5) | 1.25 | (0.98, 1.59) | 0.96 | (0.74, 1.25) | 1.20 | (0.91, 1.58) | | |
|  | Test for trend |  |  | P=0.04 |  | P=0.43 |  | P=0.14 |  | | |
| Sex | Male | 582/2173 | (26.8) | 1.00 | (ref) | 1.00 | (ref) | 1.00 | (ref) | | |
|  | Female | 1175/2904 | (40.5) | 1.88 | (1.67, 2.13) | 1.80 | (1.58, 2.06) | 2.57 | (2.18, 3.03) | | |
| Marital status  (missing=1) | Living with spouse | 1118/3483 | (32.1) | 1.00 | (ref) | 1.00 | (ref) | 1.00 | (ref) | | |
|  | Divorced/separated/widowed/never married | 639/1592 | (40.1) | 1.19 | (1.04, 1.35) | 1.07 | (0.92, 1.25) | 0.95 | (0.81, 1.11) | | |
| Lives alone  (missing=13) | No | 1520/4462 | (34.1) | 1.00 | (ref) | 1.00 | (ref) | 1.00 | (ref) | | |
|  | Yes | 232/602 | (38.5) | 1.09 | (0.91, 1.31) | 0.96 | (0.77, 1.18) | 1.02 | (0.82, 1.26) | | |
| Education | Lower than secondary | 150/377 | (39.8) | 1.23 | (0.98, 1.55) | 1.11 | (0.88, 1.40) | 1.08 | (0.85, 1.37) | | |
|  | Secondary | 964/2676 | (36.0) | 1.00 | (ref) | 1.00 | (ref) | 1.00 | (ref) | | |
|  | Tertiary | 643/2024 | (31.8) | 0.82 | (0.72, 0.93) | 0.92 | (0.80, 1.04) | 0.96 | (0.84, 1.10) | | |
|  | Test for trend |  |  | P<0.001 |  | P=0.09 |  | P=0.38 |  | | |
| Perceived financial constraints  (missing=100) | Not enough for food | 79/136 | (58.1) | 2.64 | (1.85, 3.78) | 2.47 | (1.72, 3.55) | 1.61 | (1.10, 2.37) | | |
|  | Enough for food but not clothes | 415/887 | (46.8) | 1.58 | (1.35, 1.85) | 1.53 | (1.31, 1.80) | 1.32 | (1.12, 1.57) | | |
|  | Enough for food and clothes but difficult to buy large domestic appliances | 870/2487 | (35.0) | 1.00 | (ref) | 1.00 | (ref) | 1.00 | (ref) | | |
|  | Enough for large domestic appliances but difficult to buy a new car | 297/1201 | (24.7) | 0.65 | (0.55, 0.76) | 0.67 | (0.57, 0.79) | 0.73 | (0.62, 0.87) | | |
|  | Enough for a large car | 67/266 | (25.2) | 0.68 | (0.50, 0.91) | 0.70 | (0.52, 0.95) | 0.74 | (0.55, 1.01) | | |
|  | Test for trend |  |  | P<0.001 |  | P<0.001 |  | P<0.001 | |  |  |
| Employment status  (missing=3) | In regular paid employment | 943/2985 | (31.6) | 1.00 | (ref) | 1.00 | (ref) | 1.00 | | (ref) |  |
|  | Not in regular paid employment | 814/2089 | (39.0) | 1.34 | (1.16, 1.54) | 1.18 | (1.02, 1.36) | 1.05 | | (0.90, 1.21) | |

*Model 1: Adjusted for age, sex and city ** Model 2: Model 1 + mutual adjustment for all socio-demographic factors ***Model 3: Model 2 + adjustment for smoking status, volume of ethanol, CAGE score and psycho-social factors

N for regression models= 4923

**Supplementary Table 2. Association between health behaviours and psycho-social factors and PHQ-9≥5**

|  | | PHQ-9≥5 n/N (%) | | Model 1 OR (95% CI)* | | Model 2 OR (95% CI)** | | Model 3 (95% CI)*** | |
| --- | --- | --- | --- | --- | --- | --- | --- | --- | --- |
| Smoking Status | Never Smoker | 865/2512 | (34.4) | 1.00 | (ref) | 1.00 | (ref) | 1.00 | (ref) |
|  | Ex-smoker | 369/1178 | (31.3) | 1.18 | (1.00, 1.39) | 1.19 | (1.01, 1.40) | 1.12 | (0.94, 1.33) |
|  | Current Smoker | 521/1385 | (37.6) | 1.60 | (1.37, 1.87) | 1.43 | (1.22, 1.68) | 1.23 | (1.04, 1.46) |
|  | Test for Trend |  |  | P<0.001 |  | P<0.001 |  | P=0.02 |  |
| Volume of ethanol | Non drinker | 404/1066 | (37.9) | 1.18 | (1.01, 1.38) | 1.07 | (0.91, 1.25) | 1.05 | (0.89, 1.23) |
|  | <2 Litres/year | 823/2355 | (35.0) | 1.00 | (ref) | 1.00 | (ref) | 1.00 | (ref) |
|  | 2-4.99 litres/year | 213/699 | (30.5) | 1.13 | (0.93, 1.37) | 1.14 | (0.94, 1.39) | 1.04 | (0.85, 1.28) |
|  | 5-9.99 Litres/year | 150/490 | (30.6) | 1.19 | (0.95, 1.50) | 1.19 | (0.95, 1.51) | 1.09 | (0.85, 1.38) |
|  | 10-19.99 Litres/year | 94/280 | (33.6) | 1.56 | (1.17, 2.08) | 1.58 | (1.18, 2.11) | 1.38 | (1.02, 1.86) |
|  | >20 Litres/year | 64/174 | (36.8) | 1.99 | (1.41, 2.81) | 1.75 | (1.23, 2.50) | 1.60 | (1.11, 2.31) |
|  | Test for Trend |  |  | P=0.001 |  | P=0.001 |  | P=0.03 |  |
| CAGE score among drinkers | 0 | 948/3054 | (31.0) | 1.00 | (ref) | 1.00 | (ref) | 1.00 | (ref) |
|  | 1 | 179/486 | (36.8) | 1.86 | (1.49, 2.31) | 1.83 | (1.46, 2.28) | 1.73 | (1.38, 2.17) |
|  | 2 | 131/330 | (39.7) | 2.19 | (1.70, 2.83) | 2.05 | (1.58, 2.65) | 1.78 | (1.36, 2.33) |
|  | 3-4 | 152/300 | (50.7) | 4.19 | (3.21, 5.47) | 3.74 | (2.85, 4.91) | 3.22 | (2.43, 4.26) |
|  | Test for Trend |  |  | P<0.001 |  | P<0.001 |  | P<0.001 |  |
| Enough people to confide in | Yes | 1485/4494 | (33.0) | 1.00 | (ref) | 1.00 | (ref) | 1.00 | (ref) |
|  | No | 261/563 | (46.4) | 1.88 | (1.56, 2.56) | 1.77 | (1.47, 2.14) | 1.60 | (1.31, 1.96) |
| Enough people to help when needed | Yes | 1477/4462 | (33.1) | 1.00 | (ref) | 1.00 | (ref) | 1.00 | (ref) |
|  | No | 272/590 | (46.1) | 1.81 | (1.52, 2.16) | 1.64 | (1.37, 1.97) | 1.35 | (1.11, 1.64) |
| Number of life events in the past 6 months | 0 | 652/2496 | (26.1) | 1.00 | (ref) | 1.00 | (ref) | 1.00 | (ref) |
|  | 1 | 506/1407 | (36.0) | 1.59 | (1.37, 1.83) | 1.49 | (1.28, 1.72) | 1.41 | (1.21, 1.64) |
|  | 2 | 308/669 | (46.0) | 2.32 | (1.93, 2.78) | 2.15 | (1.78, 2.58) | 1.98 | (1.64, 2.39) |
|  | 3 or more | 291/505 | (57.6) | 3.91 | (3.19, 4.81) | 3.31 | (2.68, 4.09) | 2.95 | (2.38, 3.66) |
|  | Test for trend |  |  | P<0.001 |  | P<0.001 |  | P<0.001 |  |

*Model 1: Adjusted for age, sex and city ** Model 2: Model 1 + adjustment for all socio-demographic factors ***Model 3: Model 2 + mutual adjustment for all other variables in the table (volume of ethanol and CAGE score not adjusted for each other)

N for regression models= 4923

**Supplementary Table 3. Association between demographic and socio-economic factors and Major Depression (algorithm)**

|  | | Depression n/N (%) | | Model 1 OR (95% CI)* | | Model 2 OR (95% CI)** | | Model 3 OR (95% CI)*** | | | |
| --- | --- | --- | --- | --- | --- | --- | --- | --- | --- | --- | --- |
| Age | 35-39 | 13/470 | (2.8) | 1.00 | (ref) | 1.00 | (ref) | 1.00 | (ref) | | |
|  | 40-44 | 18/650 | (2.8) | 1.01 | (0.49, 2.08) | 0.93 | (0.44, 1.94) | 0.92 | (0.43, 1.97) | | |
|  | 45-49 | 18/690 | (2.6) | 0.95 | (0.46, 1.96) | 0.95 | (0.46, 1.99) | 0.90 | (0.42, 1.93) | | |
|  | 50-54 | 24/734 | (3.3) | 1.14 | (0.57, 2.28) | 0.98 | (0.48, 1.98) | 0.88 | (0.42, 1.82) | | |
|  | 55-59 | 19/766 | (2.5) | 0.85 | (0.41, 1.75) | 0.60 | (0.28, 1.27) | 0.67 | (0.31, 1.46) | | |
|  | 60-64 | 22/857 | (2.6) | 0.87 | (0.43, 1.76) | 0.52 | (0.25, 1.09) | 0.64 | (0.29, 1.38) | | |
|  | 65-69 | 43/910 | (4.7) | 1.69 | (0.90, 3.18) | 0.92 | (0.46, 1.84) | 1.29 | (0.63, 2.66) | | |
|  | Test for trend |  |  | P=0.11 |  | P=0.38 |  |  |  | | |
| Sex | Male | 45/2173 | (2.1) | 1.00 | (ref) | 1.00 | (ref) | 1.00 | (ref) | | |
|  | Female | 112/2904 | (3.9) | 1.91 | (1.34, 2.73) | 1.77 | (1.22,2.58) | 2.37 | (1.51, 3.72) | | |
| Marital status  (missing=1) | Living with spouse | 88/3483 | (2.5) | 1.00 | (ref) | 1.00 | (ref) | 1.00 | (ref) | | |
|  | Divorced/separated/widowed/never married | 69/1593 | (4.3) | 1.51 | (1.07, 2.12) | 1.33 | (0.91, 1.95) | 1.13 | (0.77, 1.67) | | |
| Lives alone  (missing=13) | No | 134/4462 | (3.0) | 1.00 | (ref) | 1.00 | (ref) | 1.00 | (ref) | | |
|  | Yes | 22/602 | (3.7) | 1.06 | (0.66, 1.70) | 0.74 | (0.43, 1.25) | 0.84 | (0.49, 1.45) | | |
| Education | Lower than secondary | 15/377 | (4.0) | 1.28 | (0.72, 2.25) | 1.02 | (0.57, 1.83) | 0.93 | (0.51, 1.70) | | |
|  | Secondary | 89/2676 | (3.3) | 1.00 | (ref) | 1.00 | (ref) | 1.00 | (ref) | | |
|  | Tertiary | 53/2024 | (2.6) | 0.80 | (0.56, 1.14) | 0.94 | (0.66, 1.35) | 0.97 | (0.67, 1.42) | | |
|  | Test for trend |  |  | P=0.09 |  | P=0.73 |  |  |  | | |
| Perceived financial constraints  (missing=100) | Not enough for food | 21/136 | (15.4) | 7.53 | (4.39, 12.92) | 6.41 | (3.67, 11.18) | 3.30 | (1.80, 6.03) | | |
|  | Enough for food but not clothes | 48/887 | (5.4) | 2.12 | (1.43, 3.15) | 1.97 | (1.32, 2.93) | 1.55 | (1.03, 2.33) | | |
|  | Enough for food and clothes but difficult to buy large domestic appliances | 62/2487 | (2.5) | 1.00 | (ref) | 1.00 | (ref) | 1.00 | (ref) | | |
|  | Enough for large domestic appliances but difficult to buy a new car | 22/1201 | (1.8) | 0.84 | (0.51, 1.37) | 0.91 | (0.55, 1.50) | 1.12 | (0.67, 1.87) | | |
|  | Enough for a large car | 3/266 | (1.1) | 0.55 | (0.17, 1.79) | 0.60 | (0.18, 1.93) | 0.70 | (0.21, 2.29) | | |
|  | Test for trend |  |  | P<0.001 |  | P<0.001 |  |  | |  |  |
| Employment status  (missing=3) | In regular paid employment | 61/2985 | (2.0) | 1.00 | (ref) | 1.00 | (ref) | 1.00 | | (ref) |  |
|  | Not in regular paid employment | 96/2089 | (4.6) | 2.35 | (1.60, 3.45) | 1.97 | (1.33, 2.92) | 1.60 | | (1.07, 2.38) | |

*Model 1: Adjusted for age, sex and city ** Model 2: Model 1 + mutual adjustment for all socio-demographic factors ***Model 3: Model 2 + adjustment for smoking status, volume of ethanol, CAGE score and psycho-social factors

N for regression models= 4923

**Supplementary Table 4. Association between health behaviours and psycho-social factors and Major Depression (algorithm)**

|  | | Depression n/N (%) | | Model 1 OR (95% CI)* | | Model 2 OR (95% CI)** | | Model 3 (95% CI)*** | |
| --- | --- | --- | --- | --- | --- | --- | --- | --- | --- |
| Smoking Status | Never Smoker | 70/2512 | (2.8) | 1.22 | (0.76, 1.95) | 1.22 | (0.76, 1.95) | 1.11 | (0.69, 1.80) |
|  | Ex-smoker | 29/1178 | (2.5) | 1.00 | (ref) | 1.00 | (ref) | 1.00 | (ref) |
|  | Current Smoker | 57/1385 | (4.1) | 2.25 | (1.52, 3.34) | 1.81 | (1.20, 2.73) | 1.51 | (0.98, 2.33) |
|  | Test for Trend |  |  | P<0.001 |  | P=0.005 |  | P=0.07 |  |
| Volume of ethanol | Non drinker | 52/1066 | (4.9) | 1.73 | (1.19, 2.52) | 1.46 | (1.00, 2.15) | 1.37 | (0.92, 2.03) |
|  | <2 Litres/year | 69/2355 | (2.9) | 1.00 | (ref) | 1.00 | (ref) | 1.00 | (ref) |
|  | 2-4.99 litres/year | 8/699 | (1.1) | 0.52 | (0.24, 1.11) | 0.51 | (0.24, 1.09) | 0.43 | (0.20, 0.92) |
|  | 5-9.99 Litres/year | 15/490 | (3.1) | 1.53 | (0.83, 2.81) | 1.56 | (0.85, 2.89) | 1.30 | (0.69, 2.44) |
|  | 10-19.99 Litres/year | 6/280 | (2.1) | 1.18 | (0.49, 2.88) | 1.26 | (0.51, 3.08) | 1.02 | (0.41, 2.53) |
|  | >20 Litres/year | 6/174 | (3.5) | 2.07 | (0.83, 5.15) | 1.51 | (0.59, 3.86) | 1.29 |  |
|  | Test for Trend |  |  | P=0.46 |  | P=0.71 |  | P=0.44 |  |
| CAGE score among drinkers | 0 | 61/3054 | (2.0) | 1.00 | (ref) | 1.00 | (ref) | 1.00 | (ref) |
|  | 1 | 18/486 | (3.7) | 2.74 | (1.54, 4.85) | 2.50 | (1.40, 4.48) | 2.14 | (1.17, 3.91) |
|  | 2 | 19/330 | (5.8) | 4.66 | (2.63, 8.25) | 3.75 | (2.08, 6.77) | 2.85 | (1.53, 5.30) |
|  | 3-4 | 11/300 | (3.7) | 4.09 | (1.98, 8.46) | 2.88 | (1.35, 6.14) | 1.94 | (0.88, 4.29) |
|  | Test for Trend |  |  | P<0.001 |  | P<0.001 |  | P=0.004 |  |
| Enough people to confide in | Yes | 114/4494 | (2.5) | 1.00 | (ref) | 1.00 | (ref) | 1.00 | (ref) |
|  | No | 42/563 | (7.5) | 3.30 | (2.27, 4.79) | 2.76 | (1.87, 4.06) | 2.39 | (1.57, 3.65) |
| Enough people to help when needed | Yes | 122/4462 | (2.7) | 1.00 | (ref) | 1.00 | (ref) | 1.00 | (ref) |
|  | No | 35/590 | (5.9) | 2.36 | (1.59, 3.49) | 1.96 | (1.30, 2.94) | 1.18 | (0.75, 1.85) |
| Number of life events in the past 6 months | 0 | 33/2496 | (1.3) | 1.00 | (ref) | 1.00 | (ref) | 1.00 | (ref) |
|  | 1 | 39/1407 | (2.8) | 2.08 | (1.30,3.32) | 1.82 | (1.13, 2.93) | 1.73 | (1.07, 2.80) |
|  | 2 | 38/669 | (5.7) | 3.95 | (2.43, 6.40) | 3.23 | (1.97, 5.28) | 2.97 | (1.80, 4.91) |
|  | 3 or more | 47/505 | (9.3) | 7.44 | (4.67, 11.86) | 5.32 | (3.27, 8.66) | 4.72 | (2.86, 7.77) |
|  | Test for trend |  |  | P<0.001 |  | P<0.001 |  | P<0.001 |  |

*Model 1: Adjusted for age, sex and city ** Model 2: Model 1 + adjustment for all socio-demographic factors ***Model 3: Model 2 + mutual adjustment for all other variables in the table (volume of ethanol and CAGE score not adjusted for each other)

N for regression models= 4923
